# A Developmental Embodied Choice Perspective Explains the Development of Numerical Choices

**DOI:** 10.3389/fpsyg.2021.694750

**Published:** 2021-08-20

**Authors:** Alexej Michirev, Lisa Musculus, Markus Raab

**Affiliations:** ^1^Institute of Psychology, German Sport University Cologne, Cologne, Germany; ^2^School of Applied Sciences, London South Bank University, London, United Kingdom

**Keywords:** embodied choice, fingers, numerical representations, development, bounded rationality, cue, magnitude-judgment task

## Abstract

The goal of this paper is to explore how an embodied view can redirect our understanding of decision making. To achieve this goal, we contribute a developmental embodied choice perspective. Our perspective integrates embodiment and bounded rationality from a developmental view in which the body provides cues that are used in abstract choices. Hereby, the cues evolve with the body that is not static and changes through development. To demonstrate the body’s involvement in abstract choices, we will consider choices in numerical settings in which the body is not necessarily needed for the solution. For this, we consider the magnitude-judgment task in which one has to choose the larger of two magnitudes. In a nutshell, our perspective will pinpoint how the concept of embodied choices can explain the development of numerical choices.

## Introduction

Decades of theory in economics assumed *Homo sapiens* to be an agent of rationality. The surprise came when *Homo sapiens* failed to comply with these assumptions. Herbert Simon (e.g., 1972) identified those failures as the limited human ability to have complete knowledge of the world resulting in states of uncertainty. Together, the dynamic nature of the world and the limits of the human brain restrict human rationality. Simon coined these restrictions “bounds” and introduced *bounded rationality*. Half a century later, rationality is still bounded. To add to bounded rationality theorizing, we distinguish the crucial role of the body in decision making and refer to the concept of *embodied choices* (Raab, 2021).

To demonstrate embodied choices, we use the numerical setting and argue that specific body parts, such as fingers impact numerical choices; therefore, becoming *embodied* choices. Further, we consider how children use their fingers in numerical settings that create choice relevant cues, their development and impact in adulthood; therefore, taking a developmental perspective on embodied numerical choices. To assess these choices, we use the symbolic-magnitude-judgment task stemming from models of numerical cognition (for details see Knops, 2019). In these tasks, the body and its movements are not directly necessary for the choice itself, meaning that you can solve the task without an intact body, such as congenital amputees can choose among magnitudes. Showing that the body influences abstract numerical choices, therefore, would provide a strong case for the crucial role of the body, if it impacts abstract choices. Following this line of reasoning, we propose our theoretical *developmental embodied choice* (*DEC*) perspective that explains numerical choices relying on *cues* that emerged from *finger-use* and throughout *development*.

## The Three Components Constituting the Dec Perspective

### Fast-and-Frugal Heuristics: The Cues

*Fast*-and*-frugal heuristics* (Gigerenzer and Todd, 1999) are the first of three components of our *DEC perspective*. Fast-and-frugal heuristics adhere to bounded rationality and are cognitive shortcuts enabling fast choices by relying only on few task-relevant cues. Cue validity indicates how often the cue was successful in producing good or correct choices in similar situations. Thus, within bounded rationality, *we position ourselves within the fast*-and*-frugal heuristics camp* to explain choices and argue for a Homo heuristicus (Gigerenzer and Brighton, 2009) that considers the role of the body and as such constitutes our second theoretical component.

### Embodied Cognition: Finger-Use as a Cue

Presupposing bi-directionality and interdependence of body and mind, *embodied cognition* is the second component of our DEC perspective (Wilson, 2002; Barsalou, 2008; Raab, 2017, 2021). In choice settings, the body is mostly neglected because it is not regarded as a source of information that impacts choices (Raab, 2021). Assuming bi-directionality, how would the body and its processes (not) influence cognition? Here, we link fast-and-frugal heuristics to embodied cognition by considering the body as a vital cue: A concept coined embodied choices (Raab, 2017). In numerical settings, children use fingers to help them count (Butterworth, 1999). When children notice that one of their fingers corresponds to one object, they develop an understanding of the one-to-one correspondence principle (Alibali and Dirusso, 1999). In DEC, we propose that fingers are bodily cues that gain cue validity through one-to-one finger-object correspondence. Whenever the child is confronted with a choice in a numerical setting (e.g., “Am I holding one or two cards?”) it will frequently rely on its fingers and the representation thereof to choose (Butterworth, 1999). The reliance on mental representations defines the *moderate embodied cognition position* that our DEC perspective adheres to (Goldman, 2012; Raab and Araújo, 2019; overview of embodied cognition positions: Chemero, 2011; Gallagher, 2011). From this moderate position, we argue, children do not necessarily need the fingers to choose but mentally represent and use them as a cue if they made the experience that they are valid.

### Development: Finger-Use Impacts Cue Validity

Capturing experiential changes, *development* is the third and final component that we integrate into our DEC perspective. In particular, we argue that the *developing body* fuels embodied choices. Across the life span, the human body undergoes different phases of greater change, especially during childhood. During this rapid development, children fine-tune their motor and cognitive skills (Adolph and Hoch, 2019). From a developmental viewpoint, we suggest that bodily growth and motor-skill development are the foundations of cognitive development (Ridler et al., 2006; Koziol et al., 2012; Gottwald et al., 2016; Musculus et al., 2021) building the basis for learning (Adolph and Hoch, 2019). In the numerical context particularly, developmental studies highlight the positive impact of finger-use in preschool years on children’s numerical performance later in school (Fayol et al., 1998; Noël, 2005). Therefore, we argue that a developmental perspective on embodied numerical choices can help to disentangle how finger-use changes with age impacting cue validity of fingers, gestures, and hands and, thereby, numerical choices differentially.

Considering bounded rationality, embodiment, and development jointly, our DEC perspective pinpoints how the developing body and the sensorimotor system in childhood establish fingers as cues. We will make the case by re-interpreting existing studies and show that numerical representations and choices are embodied, developing throughout childhood and persisting in adulthood.

## The Showcase of Finger-Use and Numerical Performance

Rationality is as bounded as are children’s negative feelings toward mathematics. Indeed, those negative feelings can cause mathematical anxiety in and out of school (Richardson and Suinn, 1972). Approximately, 17% of the population has high math anxiety (Ashcraft and Moore, 2009), which deteriorates with age (Ma and Kishor, 1997; 2–6% in secondary-school children; Chinn, 2009) and is negatively linked to mathematical performance (Foley et al., 2017). Therefore, it is crucial to underpin and promote positive impact factors favoring numerical performance early.

Numerical performance can depend on embodied factors which make mathematics not as abstract as many believe (Lakoff and Núñez, 2000). The body, in particular, the fingers, and the use thereof play a crucial role in numerical development (Barrocas et al., 2020). Here, we focus on different aspects of finger-use in numerical settings, ranging from the use of individual fingers or hands to *finger-gnosis*, and *fine motor skills* (*FMSs*). Finger-gnosis is referred to as the ability to mentally represent your own fingers. Hereby, the experimenter touches the child’s two fingers without visual feedback and asks to identify the touched fingers (e.g., Penner-Wilger et al., 2009). FMSs capture how well one can move the fingers and are measured by motor-skill tests (e.g., Gashaj et al., 2019). A recent review summarizes the role of finger-use for preschool children’s performance in numerical tasks (Barrocas et al., 2020). The authors conclude that finger-use strongly contributes to counting, knowledge of the number system, number-magnitude processing, and calculation ability in childhood. Crucially, other domain-general cognitive processes, such as reading ability (Noël, 2005) or vocabulary (Asakawa and Sugimura, 2014), do not seem to predict numerical performance better. How is it that specific bodily based effects, such as finger-use, predict rather abstract numerical performance?

From the DEC perspective, the effects of finger-use on numerical performance provide a good showcase of embodied choice development for two reasons. First, the effects of finger-gnosis and FMSs can be tested using appropriate numerical *choice tasks*. An example of a numerical choice task is the magnitude-judgment task in which participants choose the larger of two magnitudes. Typically, magnitude-judgment tasks show the *distance effect*, that is, it is easier to distinguish two magnitudes that have a larger numerical difference between them resulting in faster and easier judgments (Dehaene et al., 1998). Moreover, performance on the magnitude-judgment task indicates magnitude representations and, therefore, conceptual understanding of magnitudes. Second, children use their fingers to count which has been shown to support their procedural and conceptual understanding of counting principles. Particularly, FMSs are linked to procedural counting skills that, in turn, contribute to conceptual knowledge (U. Fischer et al., 2018). Therefore, using fingers for numerical choices is *developmentally relevant* because it captures the transition from procedural to conceptual knowledge. Given fingers help bridge the transition from procedural to conceptual knowledge, finger-use might also aid abstract mathematical understanding. In the following, we will introduce our theoretical DEC perspective on the role of finger-use (*embodiment*) in the *development* of numerical *choices*.

## The Dec Perspective on Finger- and Hand-Use Impacting Numerical Choices

### Childhood

To illustrate our theoretical DEC perspective, first, we reinterpret the results of two exemplary longitudinal studies that depict the intra-individual development of numerical choices in children. We selected these studies because they controlled for the most neglected confounding factors regarding finger-gnosis (visual-spatial skills; Penner-Wilger et al., 2009) and FMSs (executive functions; Gashaj et al., 2019). Hereby, both studies estimated the impact of finger-use on numerical performance with a choice task, the *symbolic-magnitude-judgment task*. Second, we show that the effects of finger- and hand-use are not developmental artifacts and persist through adulthood. Third, we integrate the results of the re-interpretations in our DEC perspective.

The first study (Penner-Wilger et al., 2009) measured finger-gnosis performance by touching the children’s fingers and asking them to verbally indicate the touched finger. As children were deprived of any visual-spatial feedback, the task provided a pure assessment of children’s mental finger representations. The results showed that children whose mental finger representations were better in grade one (age 6.8 years) performed better in a symbolic-magnitude-judgment task in grade two. In particular, higher finger-gnosis indicated better numerical choices (by distance effect). Most importantly, finger-gnosis uniquely accounted for 10% of the variability in the distance effect.

For these findings, the authors themselves provided two different interpretations. First, they argued that there is a functional link between the mental representation of fingers and numbers established by finger-use to represent numerosities (Butterworth, 1999). From the DEC perspective, we share the interpretation that finger-use establishes a functional link between fingers and numbers. Outside and inside numerical settings, the repeated and practiced use of fingers results in improved finger sensitivity and motility, captured by finger-gnosis (Gracia-Bafalluy and Noël, 2008). Inside numerical settings, number representations become linked to fingers and become finger based. The quality of these finger-based representations constitutes cue validity: the higher the cue validity, the better numerical choices when such cues are used (e.g., in the magnitude-judgment task). Through the course of development, children learn that fingers are valid cues for numerical representations that help them make the correct numerical choices. Thus, we predict that the more frequent use of fingers for numerical choices will lead to higher cue validities attributed to fingers through the course of development. Alternatively, Penner-Wilger et al. (2009, p.524) offered that “the relation between finger and number representations may be one of identity, wherein the relation reflects a shared underlying representational form (Penner-Wilger and Anderson, 2008).” From the DEC perspective, we would not share this interpretation because our moderate-embodiment viewpoint suggests that we represent the body (i.e., fingers) and cognitive processes (i.e., numerical choices) separately but both can activate the other.

The second study (Gashaj et al., 2019) focused on FMSs in three tasks: threading beads, posting coins, and drawing trails (M-ABC-2; Petermann, 2009). The study showed that children with better FMSs performance in preschool (age 6.5 years) concurrently made better numerical magnitude judgments. Additionally, these children performed better in the number-line estimation task reflecting children’s understanding of magnitudes. The authors found that the two choice tasks construct a basic numerical skill, which predicted mathematical performance in grade two (age 8 years). Interestingly, there was a significant but weak relationship between FMSs and basic numerical skills (*β* = 0.31). Here, basic numerical skills strongly predicted mathematical achievement in grade two (*β* = 0.7). The authors themselves suggest that FMSs can be considered a domain-general skill that contributes to the domain-specific numerical skills (Luo et al., 2007; Cameron et al., 2016). Further, they argue that numbers have finger-based representations (Andres et al., 2007; Penner-Wilger et al., 2007) and that fingers and numbers share cortical connections (Ardila et al., 2000). The DEC perspective specifies that FMSs grant motility to fingers that enables and promotes finger-use. In numerical settings, better FMSs enhance the cue validity of fingers because finger-use gets easier (e.g., for counting and gestures). Here, DEC links FMSs and finger-gnosis and predicts that both are valid cues as basic numerical skills benefit from the ability to move the fingers individually while assigning magnitudes to fingers (Barrocas et al., 2020).

### Adolescence and Adulthood

Finger-based representations exist in children (Domahs et al., 2008) and adults (Domahs et al., 2010; Klein et al., 2011) and are therefore not restricted to a certain developmental period. In early development, children first learn to represent numerosity from 1 to 5 on one hand and then transit to represent numerosity from 6 to 10 using both hands. Such representation requires bimanual activation that is often more complex and slower than unilateral activation (Aglioti et al., 1993). Indeed, it results in a strong *five-break effect* during mental calculations in children at the age of 8.5 years showing that children deviate by exactly ±5 from the correct result (Domahs et al., 2008). Importantly, the five-break effect extends beyond childhood and is observed in westernized adults during a symbolic-magnitude-judgment task. Adults make faster choices when both numbers are represented by only one hand (e.g., a choice between 3 and 5). The choice for a set of numbers represented by two hands (e.g., 5 and 7) takes longer because the 5 is represented by one hand and the 7 by both hands (*generation hypothesis*; Domahs et al., 2010). That kind of hand-based representation occurs naturally as it splits the representations of 1–10 in two sets of fives, one for each hand. The five-break effect is systematic and strongly suggests that hand-based representations impact numerical choices. Importantly, it still persists in an adult population manifesting in mental addition (Klein et al., 2011). Together, the evidence of the five-break effect, therefore, suggests robust numerical embodiment effects of finger/hand-use and their representations that are not developmental artifacts.

**Figure 1:**
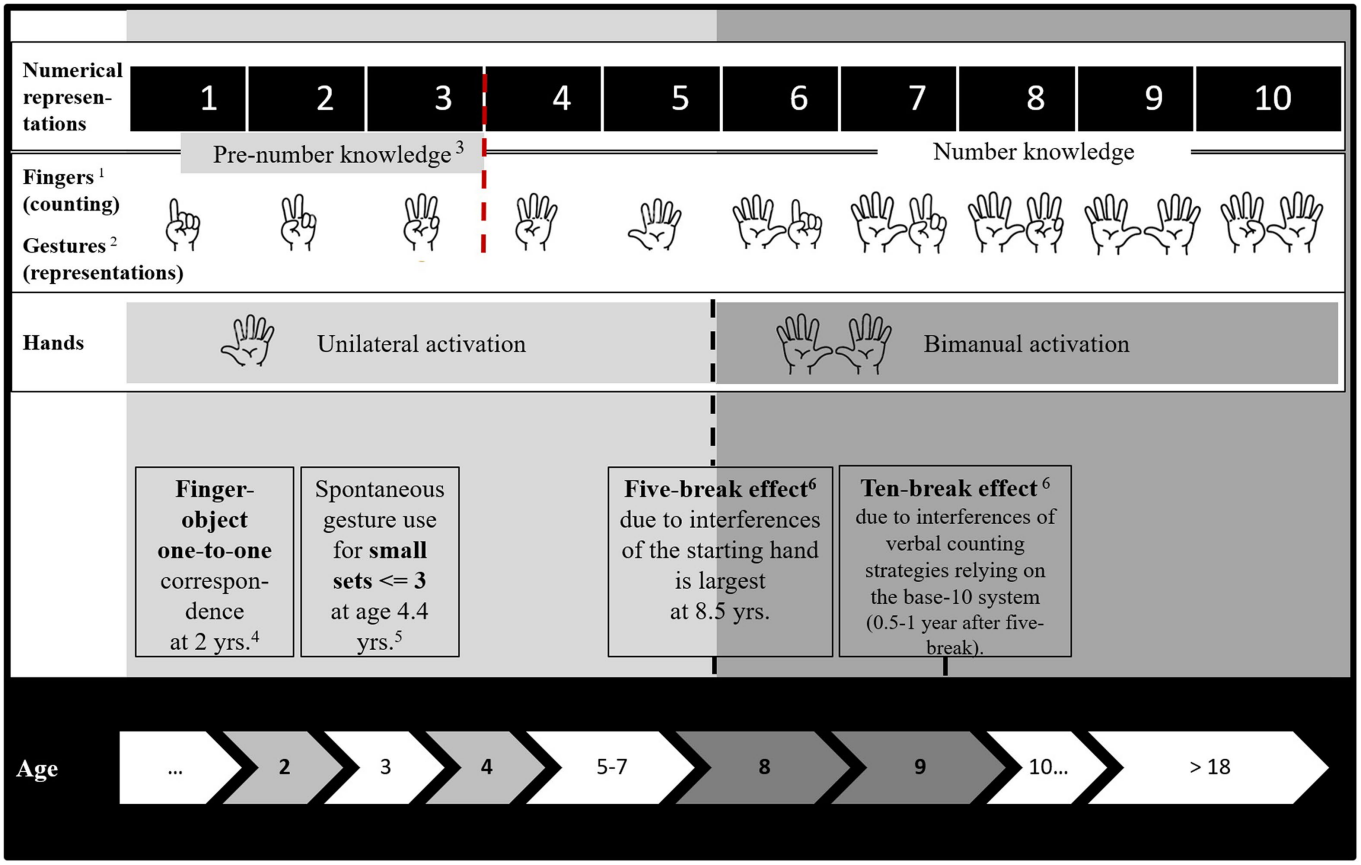
The development of finger/hand-based numerical representations that are relevant for numerical choices. The empirical evidence summarized here stems from the following references: ^1^Starting finger/hand for counting: Fischer et al., 2008; Lindemann et al., 2011; ^2^Starting finger for gesturing: Wasner et al., 2015; Spontaneous gestures: Noël, 2005; Di Luca and Pesenti, 2008; Goldin-Meadow et al., 2014; ^3^Number sense in infancy predicts mathematical performance at 3.5 years: Starr et al., 2013; ^4^Pointing gestures: Gelman and Gallistel, 1986; ^5^Accurate gesturing for sets of three and fewer: Gunderson et al., 2015; and ^6^The five-break and 10-break effects at specific ages: Domahs et al., 2008.

One interpretation of the five-break effect is that errors in working memory occur while tracking full hands (sets of fives; Domahs et al., 2008) during calculations. The second interpretation comes from the embodied cognition viewpoint and suggests that finger-based representations moderate arithmetic performance even in numerate adults (Klein et al., 2011). Considering the empirical evidence, from the DEC perspective, we predict that both fingers and hands can serve as cues and suggest the following developmental trajectory (also see Figure 1 for a conceptual summary). When children use fingers to represent sets they start with the understanding that one finger corresponds to one object (one-to-one correspondence). They proceed with counting (ordinality; counting objects in their order) and the representation of sets with gestures (cardinality; understanding that the last object in a set concludes the set; and for an overview of counting principles: Gelman and Gallistel, 1986). By the age of three, children spontaneously produce number gestures (Goldin-Meadow et al., 2014). By the age of 4.4 years, children accurately gesture sets of three or fewer (Gunderson et al., 2015). Our DEC perspective suggests that such a developmental trajectory creates particularly strong cues for the starting hand and starting finger(s) because the fingers are frequently used for counting and gesturing sets. When children learn to represent the full starting hand, the starting hand becomes a cue itself representing the entire set of five. Here, DEC proposes that the establishment of the five-break effect marks a developmental turning point. At the age of 8.5 years, when children intensively learn the mathematical base-10 system and start to count verbally, the five-break effect is particularly strong (Domahs et al., 2008). We argue from DEC that this is because the formerly established, and valid cue of the full hand (base-five) competes with the recently learned cue from the base-10 system. By the age of 8.5–9 years, the competition of base-five and base-10 diminishes and is accompanied by the increase of base-10 errors (Domahs et al., 2008). We would argue that this is another developmental turning point because verbal counting strategies (mostly) replace finger-based strategies. In conclusion, we propose that there is no reason for the five-break effect to exist if the abstract representation was not impacted by hand-based representations (Domahs et al., 2010). After all, advanced mathematical systems operate on a base-10 system, not base-five.

Taken together, we have gathered and reinterpreted evidence favoring numerical finger- and hand-based representations (Domahs et al., 2008, 2010; Penner-Wilger et al., 2009; Klein et al., 2011; Gashaj et al., 2019). From our DEC perspective, Figure 1 summarizes and illustrates the suggested developmental trajectory for finger- and hand-based representations in relation to numerical choice performance. Last, we propose future directions structured by the three components of our perspective.

## Pointing at Future Directions From the Dec Perspective

### The Heuristic Choice Component

In numerical cognition, participants are asked to make a choice. Finger-gnosis seems to correlate with magnitude judgments (e.g., Penner-Wilger et al., 2009). From an embodied choice viewpoint, it is unclear when and how bodily information is used for such choices. From our DEC perspective, we argue that if fingers are valid cues for a task then finger-gnosis or FMSs will be used in their order of validity (Gigerenzer and Todd, 1999). For this, we need to understand how finger-use manifests as a cue during development. Our DEC perspective suggests that individual finger-use (one-to-one correspondence), counting (ordinality), and gesturing (cardinality) all contribute to the cue validity of fingers. These specific time points could provide the basis for structured interventions to improve their validity.

### The Embodied Component

From an embodied cognition viewpoint, finger-gnosis and FMSs are two distinct features. The two are distinct because they might tap into different embodied choice mechanisms (Fischer and Brugger, 2011). Specifying those mechanisms that might play along the sensorimotor-cognitive continuum and to which degree finger-gnosis and FMSs share the same processes would add to future theorizing. In general, new research may want to quantify and specify the embodied effects on numerical cognition. Currently, there is a hen-egg debate whether finger-gnosis enables finger-counting or vice versa (Soylu et al., 2018). That ambiguity, and how FMSs relate to finger-gnosis and finger-counting needs to be empirically tested in cohort-longitudinal designs. Special populations can help to quantify the amount of explained variance of finger-use, finger-gnosis, and FMSs. For example, children who are born without arms and blind children who cannot rely on vision (Crollen et al., 2011) can do math. Training protocols for special-need groups that acknowledge the importance of the body may enable compensatory mechanisms for children or others at risk (Jung et al., 2015).

### The Developmental Component

Fingers, hands, and bodies, as well as their use, undergo lifelong development. While nature and nurture play their role in numerical cognition, the current mathematical education lacks clear directions. It needs to establish how the interaction of finger-gnosis/FMSs and numerical cognition is mediated by age and other individual differences (Moeller et al., 2011). Other factors, such as math anxiety (Richardson and Suinn, 1972), need to be considered because they negatively impact math performance (Foley et al., 2017). As math anxiety deteriorates with age (Ma and Kishor, 1997), preschool interventions are important. Our DEC perspective predicts that repeated use of cues should provide better cues. Therefore, interventions should start early. Interventions, such as playing with cards displaying numerosity (dots and pictures) and Arabic-symbols (mobile card game: Ponticorvo et al., 2019), could improve numerical understanding and benefit future numerical performance. Engaging in physical card games should unfold the full potential of learning because it fully engages the sensorimotor system of fingers and hands. Additionally, our DEC perspective argues that both finger-gnosis and FMSs need to be trained such that the learner is able to use this bodily information as valid cues for a choice (e.g., finger-gnosis training; Gracia-Bafalluy and Noël, 2008). It is crucial to pinpoint the time windows in which finger-gnosis and FMSs training produce the best results. Current recommendations such as longitudinal studies (Moeller et al., 2012) and investigating the timing of developmental changes (Asakawa and Sugimura, 2014) should emphasize choice mechanisms beyond executive functions (Asakawa et al., 2019).

### The Take-Home Message

The DEC perspective advocates that rationality is bounded, embodied, and affected by the developing body as well as the sensorimotor system. To pinpoint our perspective, we have considered the role of fingers and hands for numerical choices as a showcase. In sum, we propose a developmental trajectory for developmental turning points at which fingers and hands become cues (Figure 1). Cues validity increases by frequent and successful use over the course of development. We argue that at specific time points such as when the base-10 system is introduced, it builds upon our sensorimotor system (Gallese and Lakoff, 2005) and its cues. Future research should scrutinize when and how exactly the body and bodily information should be considered to improve performance in numerical and other learning environments.

## Data Availability Statement

The original contributions presented in the study are included in the article/supplementary material, and further inquiries can be directed to the corresponding author.

## Author Contributions

All authors developed the developmental embodied choice perspective and the outline of the article. AM drafted the article. LM and MR edited the article.

## Conflict of Interest

The authors declare that the research was conducted in the absence of any commercial or financial relationships that could be construed as a potential conflict of interest.

## Publisher’s Note

All claims expressed in this article are solely those of the authors and do not necessarily represent those of their affiliated organizations, or those of the publisher, the editors and the reviewers. Any product that may be evaluated in this article, or claim that may be made by its manufacturer, is not guaranteed or endorsed by the publisher.
